# Experimental Rodent Models of Cardiovascular Diseases

**DOI:** 10.3389/fcvm.2020.588075

**Published:** 2020-12-07

**Authors:** Tian Jia, Chen Wang, Zhengxi Han, Xiaozhi Wang, Ming Ding, Quanyi Wang

**Affiliations:** ^1^School of Life Science and Technology, China Pharmaceutical University, Nanjing, China; ^2^School of Pharmacy, China Pharmaceutical University, Nanjing, China; ^3^Department of Cardiology, The First Affiliated Hospital With Nanjing Medical University, Nanjing, China

**Keywords:** rodent models, cardiovascular diseases, modeling methods, coronary heart disease, hypertension

## Abstract

Cardiovascular diseases, as the most common non-communicable disease in the world, cause a high mortality rate today and bring a serious medical burden to countries worldwide, especially in low- and middle-income countries. Experimental rodent models are widely used for cardiovascular diseases researches due to the effective simulation of human cardiovascular diseases, strong reproductive ability, and easy detection. Herein, we will summarize the pathological manifestations of common cardiovascular diseases and illustrate the establishment of corresponding experimental rodent models in detail.

## Introduction

With the development of the global economy and the improvement of living and medical conditions, the extension of life expectancy and the reduction of mortality have become a continuous trend in contemporary society. The five leading causes of disability-adjusted life years (DALYs) worldwide are neonatal disease, ischemic heart disease, stroke, lower respiratory tract infections, and chronic obstructive pulmonary disease (1). As the most common non-communicable disease, cardiovascular diseases cause a large number of deaths worldwide. According to the latest data from World Health Organization (WHO), cardiovascular diseases cause 17.9 million deaths each year, accounting for about 31% of the total global deaths. More than three quarters of cardiovascular diseases deaths occur in low- and middle-income countries, bringing a particularly serious medical burden (2). Overweight and increased blood pressure and blood sugar may cause cardiovascular diseases. In addition, air pollution, smoking, unhealthy diet, and other bad habits in daily life also bring risks to heart health (3). Deep understanding of the pathogenesis and pathological characteristics of cardiovascular diseases is essential for proposing effective treatment methods or preventive measures and further reducing the prevalence and mortality.

In the research field of cardiovascular diseases, animal models play a vital role. Human atherosclerosis can be well-reproduced in rabbits and pigs. Neuberger et al. used a goat model to study chronic atrial dilation and atrial fibrillation (4). Scientists also use canine models to investigate arrhythmia or primate models to explore hypertension. However, these animal models have some disadvantages such as high experimental cost, complicated operation, high breeding environment requirements, and fewer genomic tools available (5). In contrast, rodents have stronger reproductive ability and more clear feeding conditions. Especially with excellent maneuverability and detect ability of physiological indicators, rodents have been widely used animal models for the study of cardiovascular diseases now. This article introduces the pathological basis of common cardiovascular diseases, summarizes the modeling methods of recognized rodent models, and aims to provide a reference for the research of cardiovascular diseases.

## Coronary Atherosclerotic Heart Disease

**Figure 1:**
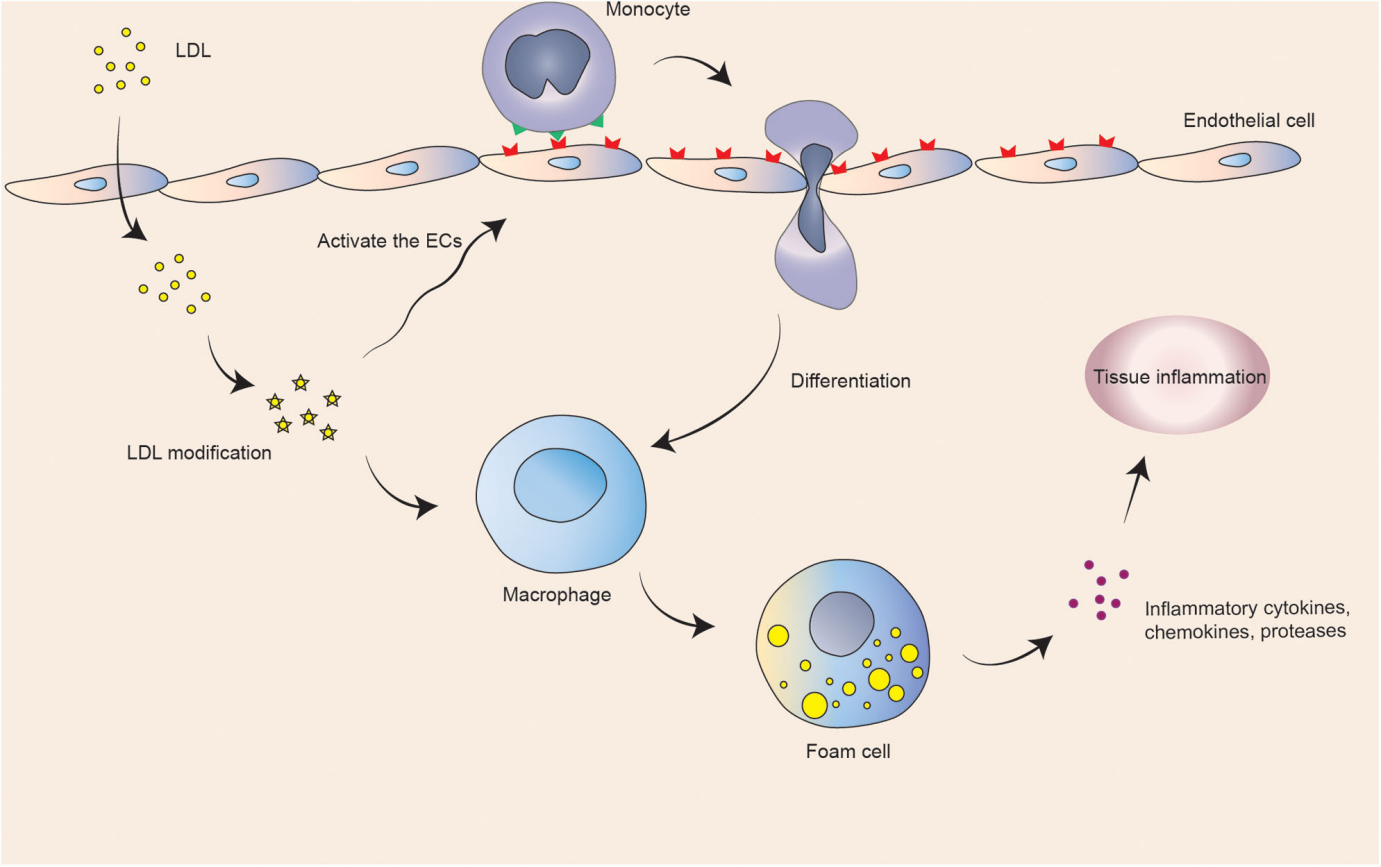
Schematic illustration of basic atherosclerotic process.

Coronary atherosclerosis is a chronic inflammatory disease that results in myocardial infarction (6). The American Heart Association (AHA) shows that an estimated 18.2 million Americans over the age of 20 suffer from coronary heart disease, with a total prevalence of 6.7%. The prevalence of men over the age of 60 is higher than that of women (7). Long-term illness may lead to persistent adverse effects on patients' work and life and increases health risks and economic burdens, making it one of the major challenges of public health worldwide (8). A large number of low-density lipoproteins that deposit on the inner wall of coronary arteries are modified to attract monocytes to recruit and differentiate into macrophages. Macrophages engulf excessive lipid substances to form foam cells and deposits in the artery, activating inflammatory reactions and eventually forming coronary atherosclerotic plaques (9). When accumulating in the plaque, redundant macrophages tend to be more likely to rupture, which may form thrombosis and cause acute myocardial ischemia. Atherosclerosis is a chronic inflammatory reaction (Figure 1). Plasma lipoproteins and inflammatory cell infiltration drive the progression of the disease. Advanced atherosclerosis exhibits an imbalance of pro-inflammatory and anti-inflammatory factors, leading to maintenance of inflammation and tissue injury (10). Clinical manifestations of coronary atherosclerosis are closely related to cardiovascular diseases, including angina, acute coronary syndrome, and heart failure (HF) (11).

Usually, mice are used as coronary atherosclerotic heart disease models. It has been reported that apolipoprotein-deficient (Apoe^−/−^) mice are more likely to develop dyslipidemia, resulting in the formation of atherosclerotic plaques. The plasma cholesterol level of Apoe-deficient mice can reach five times that of normal mice (12), showing severe hypercholesterolemia. As mice age, they are in the whole process of atherosclerosis. It is the first model that simulates human lesions well (13). The researchers analyzed the aortic plaque loaded by Sudan IV staining and the vascular lumen morphology using imaging methods and proved that Apoe^−/−^ mice fed with a high-fat diet containing 18% milk fat and 0.15% cholesterol for more than 18 weeks (14), and 12 week-old Apoe^−/−^ mice fed with a normal diet for more than 6 weeks (15), can achieve good modeling effects.

Low-density lipoprotein receptor-deficient (LDLR^−/−^) mice are another commonly used experimental model of atherosclerosis. Plaque spreads from the proximal aorta to the distal aorta over time, especially where the blood flow is disturbed (16). Plasma cholesterol levels of LDLR^−/−^ mice fed on a high fat western diet were 10 times higher than those of wild type, and plaques formed on the aortic roots, showing symptoms of atherosclerosis (17). Pan et al. fed 7 week-old male LDLR^−/−^ mice (C57BL6/J background) with a western diet containing 20% fat, 20% sugar, and 1.25% cholesterol. The mice were sacrificed after 16 weeks to prepare aortic root sections. Evaluation of atherosclerotic lesions by hematoxylin and eosin staining prove to achieve good modeling results (18). Vogel et al. fed 12 week-old male LDLR^−/−^ mice (C57BL/6 background) with a western diet containing 20% fat and 1.25% cholesterol for 8 weeks, and they stained aortic root sections with Oil Red O and Sirius red to evaluate the deposition of neutral lipids and collagen, which showed the achievement of a good modeling effect (19).

Myocardial infarction is the sequela of coronary artery disease and one of the important factors of death. It may occur during the activation of inflammation of the blood vessel wall. Additionally, in chronic diseases, myocardial infarction may worsen hemodynamics and even cause sudden death of patients. When an acute myocardial infarction occurs, the patient has diffuse discomfort in the chest, upper abdomen, and other parts for at least 20 min, normally accompanied by symptoms such as dyspnea (20). Acute necrosis of myocardial cells occurs in myocardial infarction, and fibrotic scars form during the repair. The expansion and long-term accumulation of fibrotic scars could damage the heart structure and function (21). Myocardial infarction can be modeled by left anterior descending coronary artery ligation. Aghajanian et al. used isoflurane to anesthetize male C57BL/6 mice aged 10 to 14 weeks, and they separated the fourth and fifth intercostals to expose the hearts. After the pericardium was opened, the left anterior descending coronary artery was tied using 8-0 nylon thread from the left margin of the pulmonary cone to the insertion of the left auricular appendage. This method caused permanent myocardial infarction. Four weeks later, obvious heart fibrosis was observed by Sirius red staining (22). Park et al. performed inhalation anesthesia on male Fischer 344 rats (180–200 g) with 2% isoflurane and mechanically ventilated them with medical oxygen. After the left thoracic cage was shaved and cut, a sterile polyethylene glycol tube was used to bind the suture to the left anterior descending coronary artery for 1 min and then suture with the 7-0 pro suture. Eight weeks after the operation, the rat hearts were evaluated using the Masson staining method, which was proved to achieve a good modeling effect (23).

## Hypertensive Heart Disease

A retrospective observational study showed that hypertensive heart disease displayed a negative impact on the life expectancy of human in many countries (24). Based on WHO statistics, an estimated 1.13 billion people worldwide suffer from hypertension, but less than one fifth of hypertensive patients have received effective treatment. Two thirds of hypertension patients live in low- and middle-income countries. Obesity, glucose intolerance, and dyslipidemia are often accompanied by high blood pressure, leading to the occurrence of the coronary heart disease and peripheral artery disease. Long-term hypertension may eventually lead to HF and progressive renal failure (25). Hypertensive patients have a higher incidence of sudden cardiac death with left ventricular hypertrophy (26).

Common rodent models of hypertensive heart disease include low-renin hypertension and spontaneous hypertension. The renin–angiotensin–aldosterone system is essential for maintaining blood pressure. Renal secretion in renal hypotonic state activates the system and eventually produces angiotensin II, which in turn leads to the adrenal gland to secrete aldosterone to maintain blood pressure (27). As the disease duration increases, it may be accompanied by coronary artery diseases, left ventricular hypertrophy, and HF (28). Aldosterone/salt therapy (ALDOST) was used as the modeling of low renin hypertension (29). Eight week-old male Sprague–Dawley rats were injected subcutaneously with angiotensin II (9 μg/h) via a micro pump for 2 weeks or aldosterone (0.75 μg/h) via a micro pump for 6 weeks. All of the rats showed ventricular fibrosis, and the volume fraction of interstitial collagen increased significantly, and both atria of rats treated with angiotensin II showed slight scarring (30).

The occurrence of hypertension is due to the combination of environmental factors, genetic factors, and risk behaviors. Essential hypertension with undetermined etiology accounts for ~90% of the total cases (31). Spontaneously hypertensive rats (SHRs) are the pure line isolated from Wistar rats, which spontaneously showed stable hypertension symptoms in inbreeding offspring and developed high blood pressure at 7–15 weeks of age (32). Through histopathological examination, Huc et al. found that 16 week-old SHRs showed cardiomyocyte hypertrophy and coronary artery smooth muscle cell hypertrophy. At the age of 60 weeks, rats showed increased left ventricular wall thickness, coronary fibrosis, and impaired cardiac diastolic function (33). SHR model made a valuable contribution to the study of the genetic mechanism of human essential hypertension (34).

## Heart Failure

Currently, HF is the main cause of hospitalization for adults and a risk factor for postoperative death, which has brought a rapidly increasing burden to the global public health system (35). As shown from AHA data, an estimated 6.2 million Americans over the age of 20 suffer from HF. It is predicted that from 2012 to 2030, the prevalence of HF will increase by 46%, with more than 8 million patients over 18 years old, and the total percentage will increase from 2.42% in 2012 to 2.97% in 2030 (7). HF leads to cardiac dysfunction, accompanied by various persistent symptoms such as dyspnea, edema, and fatigue, which significantly affect the patient's quality of life (36). HF is a secondary disease of various cardiovascular diseases, such as coronary artery disease and dilated cardiomyopathy. And it is also related to obesity and other systemic diseases. It usually presents with impaired left ventricular function, and reduced or preserved ejection fraction (37). The ischemic injury caused by acute myocardial infarction activates the inflammatory response through active oxygen and complement cascade reaction and promotes the recruitment of immune cells to ischemic myocardium. These series of reactions is closely related to HF (38).

Cho et al. used Dahl salt-sensitive (DSS) rats to model the ejection fraction-preserving HF. Seven week-old male DSS rats were fed a high-salt diet (containing 8% NaCl), and most of them showed symptoms of HF at 14 weeks of age. Echocardiography was used to measure the systolic and diastolic function of the heart at 14 and 18 weeks. Rats with diastolic dysfunction but retained systolic function were considered successful in modeling (39). Additionally, mice undergoing transverse aortic constriction (TAC) surgery showed cardiac hypertrophy after 5 days, attenuated ejection fraction after 14 days, and HF after 30 days (40). Greco et al. used 2 month-old male C57BL6/J mice to numb a mixture of ketamine (100 μg/kg) and xylazine (10 μg/kg) for anesthesia. The aortic arch was exposed to the first intercostal space, 8-0 suture was tied to a 27-gauge needle for narrowing, and the chest cavity was sutured with 6-0 silk thread. The pressure load caused by TAC surgery was verified by echocardiography (41). Chronic isoproterenol stimulation of mice was used to mimic human advanced HF. Wang et al. used 8 to 10 week-old infertile female mice in the mixed mouse diversity group, anesthetized the mice by intraperitoneal injection of ketamine, and implanted micro pump into the peritoneum of mice. The dose of isoproterenol is 30 mg/kg body weight per day for 21 days. Weekly echocardiography examination showed significant changes in the size and quality of the left ventricle, demonstrating good modeling effect (42).

## Rheumatic Heart Disease

Rheumatic heart disease is a crucial preventable cause of death and disability caused by cardiovascular diseases. Due to the impact of medical conditions and living environment, rheumatic heart disease still has a high incidence in developing countries. The new meta-analysis from WHO data illustrated that the prevalence of rheumatic heart disease was 11.3%, and ~2% of deaths from cardiovascular disease are related to rheumatic heart disease (43). In Africa, South Asia, and many other regions, the high mortality rate of rheumatic heart disease cannot be ignored (44). Rheumatic heart disease was mainly caused by group A β-hemolytic streptococcal infection, which triggered an autoimmune response against collagen (45). Rheumatic heart disease may be complicated by endocarditis, atrial fibrillation, and thromboembolic stroke (46), resulting in damages to the heart valves and left ventricular abnormalities, and ultimately chronic HF and death (47).

Previous research by Quinn et al. showed that group A β-hemolytic streptococcus M5 protein induced rat autoimmune valvulitis, which can be used as an animal model for the rheumatic heart disease (48). Gorton et al. reported that female Lewis rats aged 8–12 weeks were injected subcutaneously with 0.5 mg of rM5 or M5 peptide emulsified 1:1 with complete Freund's adjuvant. On days 1 and 3, 1 × 10^10^ completely inactivated *Bordetella pertussis* was intraperitoneally injected to promote Th1 autoimmune response. On day 7, the same dose of rM5 or M5 peptide was used to boost the immunization subcutaneously in the ventral side. On day 21, the results of indirect sterilization tests showed that rM5 could cause Lewis rats to produce opsonized antibodies against group A streptococci. In the presence of immunized rat serum, the number of colonies forming units in non-immunized human blood was reduced by more than 98%. The production of opsonization antibodies and the inflammatory infiltration of heart tissues of CD3^+^, CD4^+^, and CD68^+^ T cells proved good modeling effect (49).

Another report revealed that group A streptococci were inactivated with formaldehyde and made into a suspension with physiological saline. The solution was completely emulsified by sonic disruption and added with an equal volume of complete Freund's adjuvant to prepare antigen A and B, respectively. Eight week-old female Lewis rats were injected with antigen. On day 0, 0.2 ml of antigen B was injected into the hind footpad. On days 7, 14, 21, and 28, 0.5 ml of antigen B was injected subcutaneously into the abdomen. On days 35, 42, 49, and 56, antigen A was injected subcutaneously. On day 63, the rats showing heart valves injury and fibrosis were successful rheumatic heart disease models (50).

## Pulmonary Heart Disease

Pulmonary heart disease is closely related to pulmonary hypertension. Mild pulmonary hypertension is often associated with chronic lung disease, and severe hypertension is related to the right ventricular dysfunction. Pulmonary hypertension can be induced by drugs, toxins, and genes. WHO divides pulmonary hypertension into five groups based on the underlying pathogenesis. Pulmonary hypertension patients caused by genetic, drug/toxin induction, infection, and other factors are 6.6 to 26.0 per million adults per year, and the incidence rate is 1.1 to 7.6 per million adults per year. The common cause is the mutation of bone morphogenetic protein type 2 receptor (BMPR2), a member of transforming growth factor (TGF)-β superfamily (51). Hypertrophic remodeling occurs in the right ventricle of patients with pulmonary heart disease and may be accompanied by various chronic respiratory diseases. It results in insufficient arterial oxygen content, increased heart rate, and cardiac output. Progressive right ventricular failure is an important cause of death late in the course of the disease (52).

Some researchers have found that injection of monocrotaline combined with chronic hypoxic environment induces pulmonary hypertension in Wistar rats. Seven days after the administration of monocrotaline, the rats exhibited muscularization of the pulmonary arteries. Ten days later, pulmonary hypertension appeared, and 12 days later, the right ventricular hypertrophy could be detected (53). Coste et al. injected monocrotaline (60 mg/kg) intraperitoneally into male Wistar rats (250–350 g) and then raised them in a hypoxic hypobaric chamber (380 mmHg) for 28 days. Magnetic resonance imaging showed a significant decrease in right heart ejection fraction, and hemodynamic changes showed a significant increase in right systolic blood pressure (54). Dai et al. injected monocrotaline (32 mg/kg) into 6 week-old male Sprague–Dawley rats. After 28 days, the echocardiography and right ventricular hemodynamic evaluation revealed hypertrophic right ventricle and impaired contractility (55).

## Myocarditis

Myocarditis is a common cardiovascular disease with clinical manifestations including arrhythmia and severely impaired cardiac function. The main consequence of persistent myocardial inflammation is dilated cardiomyopathy, accompanied by chronic HF. The probability of new ventricular tachycardia events in patients with myocarditis is higher than that of healthy people (56). The viral infection is the most common cause of myocarditis. In the early stage of infection, the virus causes cardiomyocyte death through apoptosis and autophagy, activating the immune response. In the late infections, a large number of immune cells accumulate in the infected heart, and the expression of pro-inflammatory cytokines is significantly elevated, resulting in severe heart damage (57). Idiopathic giant cell myocarditis is a rare autoimmune disease that usually develops rapidly and has a high mortality rate (58). It is characterized by the appearance of multinucleated giant cells, inflammatory infiltration of lymphocytes, and myocardial cell necrosis (59).

The recognized model of viral myocarditis is induced by Coxsackie virus B3 (CVB3) (60, 61). Six week-old male specific-pathogen-free (SPF) mice were injected intraperitoneally with 0.1 ml of CVB3 dilution, and the virus titer was 100 TCID50 (50% tissue culture infection dose)/0.1 ml. After 7 days of consecutive injection, the peripheral blood and heart were collected on the eighth day. Cardiac inflammatory cell infiltration, myocardial necrosis ratio, expression of tumor necrosis factor-α (TNF-α), and TGF-β1 proved that the modeling effect was good (62). In addition, 1.0 × 10^6^ plaque-forming units of purified CVB3 were diluted in 0.1 ml of phosphate buffer and injected intraperitoneally into 4 week-old male BALB/c mice. On day 7, the modeling effect was evaluated by cardiomyocyte apoptosis (63).

A mouse model of experimental autoimmune myocarditis can be induced by the α-myosin heavy chain (α-MHC) (64). Diny et al. injected intraperitoneally 500 ng of pertussis toxin into male BALB/c mice aged 6 to 10 weeks on day 0. On days 0 and 7, mice were subcutaneously immunized with 100 μg of α-MHC_614−629_ peptide emulsified with complete Freund's adjuvant and 5 mg/ml of heat-inactivated tuberculosis branches *Bacillus* H37Ra. On day 21, flow cytometry was used to evaluate the inflammatory cell infiltration in the heart, and the degree of fibrosis was observed by Masson staining, which proved that a good modeling effect was achieved (65). It was also reported that 8 week-old male BALB/c mice were injected subcutaneously with 200 μg of α-MHC synthetic peptide emulsified with complete Freund's adjuvant on days 1 and 7. On day 21, the hearts were analyzed by echocardiography and flow cytometry, and the ideal modeling effect was obtained (66).

## Congenital Heart Disease

Congenital heart disease is the most common cause of congenital abnormalities in neonates. During embryonic development, nearly one third of congenital diseases are manifested as serious defects in the cardiac structure and function. Each year, heart malformations affect at least 2% of newborns worldwide, and long-term medical needs cause high fetal mortality. In the United States, it is estimated that one in 150 adults will have congenital heart disease. In Canada, the expected growth rate of congenital heart disease is 1 to 5% per year (7). Children born in developing countries have a higher proportion of congenital heart disease (67). The types of congenital heart disease include arterial stenosis, atrial septal defect, and ventricular septal defect (68).

Researches on the genetic mechanism of congenital heart disease are still in progress, and there are still a considerable number of cases that are difficult to explain. It is estimated that there are as many as 400 genes related to congenital heart disease. Mutations in transcription factors and cell signal transducers that are closely related to heart development lead to the loss of heart structure and abnormal function. The functional network formed by the proteins encoded by these genes may also be related to the occurrence and development of congenital heart disease (69).

Evolutionary highly conserved homeobox transcription factor Nkx2-5 is essential for the heart development. Nkx2-5 defects caused abnormal defects such as dysplasia, conduction block, and ventricular septal malformation (70). The mouse Nkx2-5 protein undergoes a point mutation from I to M or I to P at residue I183, resulting in a change in 183 at methionine or proline residues. After two heterozygous mice were crossed, severe growth retardation and cardiac abnormalities were observed in the embryos of homozygous offspring (71). Studies have also shown that the introduction of missense mutations in the homology domain 52 and 188 position of Nkx2-5 protein in 129/Sv mice, Arg52 (188) Gly, can result in heterozygous knock-in mouse model Nkx2-5^+/R52G^. The cardiac malformation of knock-in mice affects the cardiac morphology and function, showing the ventricular septal defect, atrioventricular septal defect, and tricuspid valve abnormalities. It imitated the persistent room communication in human congenital heart disease, which could be used as an animal model of the progressive atrioventricular block (72).

The transcription factor GATA4 participates in the development of the heart. GATA4 is involved in regulating the expression of the α-MHC, cardiac troponin C, and other structural genes. GATA4-deficient mice have severe ventricular development defects, leading to heart malformation and embryonic death. GATA4 M310V transgenic C57BL/6 mice (methionine to valine amino acid site-specific mutations) were used as the atrial septal defect model. The effect of modeling can be evaluated by detecting the cardiac structure and function of heterozygous mice (73).

## Vascular Disease

Coronary artery calcification exists and is accompanied by the development of advanced coronary atherosclerosis (74). High blood pressure, diabetes, hyperphosphatemia, and hypercalcemia caused by renal dysfunction all result in coronary artery calcification (75). Many long-term observational studies based on the population have shown that there is a close relationship between coronary artery calcification and cardiovascular diseases. Coronary artery calcification score can improve the cardiovascular risk assessment of asymptomatic individuals and advise the timing of preventive treatment (76). Osteoprotegerin (OPG) is linked to angiogenesis and plays an important role in protecting arteries from pathological calcification (77). OPG knockout (OPG^−/−^) mouse is a widely used model of arterial calcification. OPG^−/−^ mice were generated by replacing the 279-bp region of OPG exon 2 with a PGK-neo box. Computed tomography confirmed that 12 week-old OPG^−/−^ mice showed significant calcium loss in the spine and obvious calcification of the aorta and heart (78).

Maintaining normal hemodynamics is important for internal circulation. Vascular calcification impairs vasodilation and increases the risk of vascular rupture. It may cause left ventricular hypertrophy, HF, myocardial infarction, and other adverse consequences (79). Vascular calcification includes medial calcification and intimal calcification. Chronic kidney disease-induced hyperphosphatemia may result in mineral imbalance, accelerate calcium phosphate deposition, and develop severe intravascular calcification, which greatly increases the probability of death due to cardiovascular lesions (80). Adenine is commonly used for modeling chronic kidney disease. Male Wistar rats (160–180 g) were gavage with adenine at a dose of 250 mg/kg/day for 2 weeks and then at a dose of 250 mg/kg every other day for 4 weeks. Six weeks later, the rats were sacrificed. Alizarin red S staining and von Kossa staining of the artery proved that the rats showed obvious medial calcification (81). Giachelli et al. performed a two-step surgical procedure for partial renal ablation on 18 week-old female dilute brown non-agouti (DBA/2) mice. First, the right kidney was exposed, decapsulated, and partially electrocauterized. Two weeks later, the left kidney was completely removed. After 72 h, the mice were fed a high-phosphate diet containing 0.9% phosphate and 0.6% calcium. After 12 weeks, the aorta was analyzed, and Alizarin red-stained sections proved that the mice showed medial calcification (82). Jahnen-Dechent et al. fed fetuin-A/apolipoprotein E-deficient (Ahsg^−/−^/Apoe^−/−^) C57BL/6 mice with a high-phosphate diet containing 1.65% phosphate, 0.95% calcium, 4.5% fat, and 17% protein. The mice were sacrificed 9 weeks later. Von Kossa staining analysis of aortic sections showed that the mice showed obvious vascular calcification, and most of them appeared in the intimal area (83). Vascular calcification is caused by the differentiation of vascular smooth muscle cells into osteoblast-like cells. The expression of Runt-related transcription factor 2 (RUNX2) increases significantly during vascular remodeling and calcification and plays an important role in this pathological process (84). Giachelli et al. generated mice that SM22Cre-directed removal of Runx2 in blood vessels (LDLR^−/−^: Runx2^Δ*SM*^) and fed them with a high-fat diet including 1.25% cholesterol, 39.9% kcal fat, and 40% kcal carbohydrate. After 18–24 weeks, Movat pentachrome staining showed that loss of SMC-specific Runx2 reduces arterial intimal calcification without affecting the size of atherosclerotic lesions (85).

Kawasaki disease is an acute vasculitis syndrome with symptoms including acute fever, mucosal inflammation, and rash. It is the most common vascular inflammation in children (86). Kawasaki disease mainly affects small and medium arteries, especially coronary arteries, which may lead to aneurysm formation and thrombosis, causing myocardial ischemia and even death (87). Studies have shown that the water-soluble extract of *Candida albicans* (CAWS) induced vasculitis in the roots and coronary arteries of mice. C57BL/6 mice were intraperitoneally injected with CAWS (1 mg) once a day for 5 days. Coronary heart slices analysis demonstrated that the inflammatory cells were recruited to the root of the aortic, thus proving good modeling effects (88). In addition, the mouse Kawasaki disease model induced by *Lactobacillus casei* cell wall extract (LCWE) reflected the pathological features of human Kawasaki disease such as coronary arteritis and coronary artery stenosis (87). A single dose of 500 μl of LCWE was injected intraperitoneally into male C57BL/6 mice aged 4 to 5 weeks. Hematoxylin and eosin staining was used to assess the heart inflammation on days 7, 14, and 35 after injection to test the effectiveness of modeling (89).

## Conclusions and Future Perspectives

As the heart is an organ that begins to form in early embryonic development, cardiac development involves the co-regulation of multiple morphogenetic systems and the interaction between various cell populations. Diverse signaling molecules regulate cardiac development by transcription factors, which is extremely complex. The chronically poor living environment brings the risk of cardiovascular disease. Long working hours (90), high occupational noise (91), and traffic pollution (92) all have negative effects on the cardiovascular system. The research on the pathogenesis and treatment of human cardiovascular diseases could benefit from modeling technological advances.

Large animal models such as canine models and primate models have a long history of being used in cardiovascular diseases researches. Although they are good at simulating the pathological characteristics of human patients, there are inevitable limitations including high feeding costs and difficulties in genetic modification. Undoubtedly, rodents are the most common cardiovascular disease model, which are the indispensable tools for researches on the pathological features, clinical symptoms, and drug development of human diseases. Rodent models not only effectively simulate characteristics and indicators of human cardiovascular diseases but also have advantages of strong reproductive ability and easy detection, providing great convenience for scientific researches. For coronary atherosclerotic heart disease, hypertension heart disease, HF, myocarditis, and other common cardiovascular diseases introduced in this article, recognized rodent models have been developed and widely used (Table 1). These models reliably simulate various characteristics and indicators of human cardiovascular diseases and make immense contribution for scientific researches (Table 2). However, experimental rodent models are very demanding. Some existing rodent models for cardiovascular diseases have not been mature and effective, such as coronary aneurysms. Coronary aneurysms exhibit a variety of clinical symptoms, including angina pectoris, an acute coronary syndrome in the presence of obstructive atherosclerosis, and myocardial infarction caused by local thrombosis. Its onset is mainly affected by atherosclerosis and Kawasaki disease (95). Further coronary aneurysm researches depend on the constantly improved rodent models.

**Table 1:** The common cardiovascular diseases and the modeling methods of recognized rodent models.

**Model classification**	**Cardiovascular diseases**	**Modeling methods**	**Advantage and limitation**
Genetic models	Coronary atherosclerotic heart disease	Apoe^−/−^ mice	Uniform phenotype, good controllability, long breeding time, high cost
	Essential hypertension	Spontaneously hypertensive rats (SHRs)	
	Congenital heart disease	Nkx2-5 defect mice, GATA4 defect mice	
	Coronary artery calcification	OPG^−/−^ mice	
	Arterial intimal calcification	LDLR^−/−^: Runx2^Δ*SM*^ mice	
Special diet models	Coronary atherosclerotic heart disease	Feed Apoe^−/−^ mice with high-fat diet, feed LDLR^−/−^ mice with high-fat diet	Low cost of food, simple operation, large individual differences, long experiment period
	Ejection fraction-preserving heart failure	Feed Dahl salt-sensitive (DSS) rats with high-salt diet	
	Coronary medial calcification Coronary intimal calcification	Feed DBA/2 mice with high-phosphate diet Feed Ahsg^−/−^, Apoe^−/−^ mice with high-phosphate diet	
Surgical induced models	Myocardial infarction	Left anterior descending coronary artery ligation	Significant effect, difficult operation, high technical requirements
	Heart failure	Transverse aortic constriction (TAC) surgery	
Drug or immunogen-mediated models	Low renin hypertension Advanced heart failure	Aldosterone/salt therapy (ALDOST) Chronic isoproterenol stimulation	Significant effect, simple operation, large individual differences, risk of infecting operators
	Rheumatic heart disease	Group A β-hemolytic streptococci	
	Pulmonary heart disease	Injection of monocrotaline combined with chronic hypoxic environment	
	Viral myocarditis	Coxsackie virus B3 (CVB3)	
	Experimental autoimmune myocarditis	α-Myosin heavy chain (α-MHC)	
	Coronary medial calcification	Adenine	
	Kawasaki disease	Water-soluble extract of *Candida albicans* (CAWS), *Lactobacillus casei* cell wall extract (LCWE)	

**Table 2:** Rodent models and important findings.

**Rodent models**	**Important findings**
Apoe^−/−^ mice	Interleukin-1β against late atherosclerosis in mice (14) Targeting CD40-induced TRAF6 signaling in macrophages reduces atherosclerosis (15)
LDLR^−/−^ mice with high-fat diet	Folic acid delays development of atherosclerosis in LDLR^−/−^ mice (18) Bilirubin prevents atherosclerotic lesion formation in LDLR^−/−^ mice (19)
Left anterior descending coronary artery ligation model	Redirected T cell immunotherapy can be used to treat pathological cardiac fibrosis in mice (22) Dual stem cell therapy improves cardiac function and vascular regeneration after myocardial infarction (23)
Spontaneously hypertensive rats (SHRs)	Chronic, low-dose TMAO treatment reduces diastolic dysfunction and heart fibrosis in hypertensive rats (33)
Transverse aortic constriction (TAC) surgery model	DNA hydroxymethylation controls cardiomyocyte gene expression in development and hypertrophy (41)
Dahl salt-sensitive (DSS) rats	Delayed repolarization underlies ventricular arrhythmias in rats with heart failure and preserved ejection fraction (39)
Group A β-hemolytic streptococci induced rheumatic heart disease model	Rats immunized with streptococcal M5 protein developed valvular lesions, rheumatic fever, and rheumatic heart disease are mediated by inflammatory CD4^+^ T cells and CD68^+^ macrophages (49) Inhibition of miR-155-5p attenuates the valvular damage induced by rheumatic heart disease (50)
Monocrotaline-induced pulmonary heart disease model	Inhibition of HIF-2α can be used to treat severe vascular remodeling and right heart failure in pulmonary arterial hypertension (55)
Coxsackie virus B3 (CVB3) induced myocarditis model	Nicotinic agonist inhibits cardiomyocyte apoptosis in CVB3-induced myocarditis (63)
α-Myosin heavy chain (α-MHC) induced autoimmune myocarditis model	Eosinophil-derived IL-4 drives progression of myocarditis to dilated cardiomyopathy (65) Midkine drives cardiac inflammation by promoting neutrophil trafficking and NETosis in myocarditis (66)
Nkx2-5 defect mice	Point mutations in murine phenocopy human congenital heart disease and induce pathogenic Wnt signaling (71)
GATA4 defect mice	Cardiac-specific deletion of Gata4 reveals its requirement for hypertrophy, compensation, and myocyte viability (93)
OPG^−/−^ mice	MicroRNA-32 promotes calcification in vascular smooth muscle cells (78)
Adenine-induced coronary medial calcification model	Ginsenoside Rb1 ameliorates chronic kidney disease (CKD)-associated vascular calcification by inhibiting the Wnt/β-catenin pathway (81)
LDLR^−/−^: Runx2^Δ*SM*^ mice	Runx2 deletion in smooth muscle cells inhibits vascular osteochondrogenesis and calcification but not atherosclerotic lesion formation (85)
Water-soluble extract of *Candida albicans* (CAWS) induced Kawasaki disease	Dectin-2-induced CCL2 production in tissue-resident macrophages ignites cardiac arteritis (88)
*Lactobacillus casei* cell wall extract (LCWE) induced Kawasaki disease	Interleukin-1β is crucial for the induction of coronary artery inflammation in a mouse model of Kawasaki disease (94)

Although some progress has been made in this field, we still face various difficulties. For example, Cui et al. analyzed the single-cell transcriptomes of human and mouse fetuses and confirmed difference in gene expression profiles between the two. Cardiomyocytes were not synchronized in mouse and human embryo development (96). In addition, because of longer pregnancy, the occurrence time and pathological manifestations of heart defects, such as left heart hypoplasia syndrome, may be different in human and mouse fetuses (97). More effort is needed to exploit more suitable experimental rodent models for human cardiovascular diseases.

## Author Contributions

CW, ZH, XW, MD, and QW contributed to the conception and design of the review. TJ collected data and wrote the manuscript. All authors contributed to manuscript revision and read and approved the submitted version.

## Conflict of Interest

The authors declare that the research was conducted in the absence of any commercial or financial relationships that could be construed as a potential conflict of interest.
